# Enhanced Language Models for Predicting and Understanding HIV Care Disengagement: A Case Study in Tanzania

**DOI:** 10.21203/rs.3.rs-6608559/v1

**Published:** 2025-05-08

**Authors:** Waverly Wei, Junzhe Shao, Rita Qiuran Lyu, Rebecca Hemono, Xinwei Ma, Joseph Giorgio, Zeyu Zheng, Feng Ji, Xiaoya Zhang, Emmanuel Katabaro, Matilda Mlowe, Amon Sabasaba, Caroline Lister, Siraji Shabani, Prosper Njau, Sandra I. McCoy, Jingshen Wang

**Affiliations:** University of Southern California; University of California, Berkeley; University of California, Berkeley; University of California, Berkeley; University of California, San Diego; University of California, Berkeley; University of California, Berkeley; University of Toronto; University of Florida; Health for a Prosperous Nation, Dar es Salaam, Tanzania; Health for a Prosperous Nation, Dar es Salaam, Tanzania; Health for a Prosperous Nation, Dar es Salaam, Tanzania; Dodoma Referral Hospital, Dodoma, Tanzania; Ministry of Health, Dodoma, Tanzania; Ministry of Health, Dodoma, Tanzania; University of California, Berkeley; University of California, Berkeley

## Abstract

**Summary:**

Sustained engagement in HIV care and adherence to antiretroviral therapy (ART) are essential for achieving the UNAIDS “95-95-95” targets. Despite increased ART access, disengagement from care remains a significant issue, particularly in sub-Saharan Africa. Traditional machine learning (ML) models have shown moderate success in predicting care disengagement, which would enable early intervention. We develop an enhanced large language model (LLM) fine-tuned with electronic medical records (EMRs) to predict people at risk of disengaging from HIV care in Tanzania and to provide interpretative insights into modifiable risk factors.

**Methods:**

We developed a novel AI model by enhancing a pre-trained LLM (LLaMA 3.1, an open-source pre-trained LLM released by Meta) using routinely collected EMRs from Tanzania’s National HIV Care and Treatment Program from January 1, 2018, to June 30, 2023 (4,809,765 records for 261,192 people) to identify people at risk of disengaging from HIV care or developing adverse outcomes. Outcomes included risk of ART non-adherence, non-suppressed viral load, and loss to follow-up. Models were evaluated internally (Kagera region) and externally (Geita region), with performance compared against state-of-art ML models and zero-shot LLMs. Additionally, a team of HIV physicians in Tanzania assessed the LLM’s predictions along with LLM provided justifications for a subset of patient records to evaluate their clinical relevance and reasoning.

**Findings:**

The enhanced LLMs consistently outperformed the supervised ML model and zero-shot LLMs across all outcomes in both internal and external validation datasets. When focusing on the 25% of PLHIV predicted as most likely to lost-to-follow-up (LTFU), the model correctly identified 78% (2,515 of 3,224) of people living with HIV (PLHIV) genuinely at risk in internal validation and 73% (7,105 of 9,733) in external validation. Attention score analysis indicated that the enhanced LLM focused on keywords such as gaps in follow-up care and ART adherence. The human expert evaluation showed alignment between clinician assessments and the LLM’s predictions in 65% of cases, with experts finding the model’s justifications reasonable and clinically relevant in 92.3% of aligned cases.

**Interpretation:**

If implemented in HIV clinics, this LLM-based AI model could help allocate resources efficiently and deliver targeted interventions, improving retention in care and advancing the UNAIDS “95-95-95” targets. By functioning like a clinician–analyzing patient summaries, predicting risks, and offering reasoning–the enhanced LLM could be integrated into clinical workflows to complement human expertise, facilitating timely interventions and informed decision-making. If implemented widely, this human-AI collaboration has the potential to improve health outcomes for people living with HIV and reduce onward transmission.

**Funding:**

The study was supported by a grant from the US National Institutes of Health (NIH): NIH NIMH 1R01MH125746.

## Introduction

The global effort to control the HIV epidemic critically depends on sustained engagement in care and adherence to antiretroviral therapy (ART). The Joint United Nations Programme on HIV/AIDS (UNAIDS) has set ambitious “95-95-95” targets for 2030,^1^ aiming for 95% of people living with HIV (PLHIV) to know their status, 95% of those diagnosed to receive ART, and 95% of those on ART to achieve viral suppression. Central to these goals is the widespread and effective use of ART, which not only improves the quality of life for PLHIV but also reduces the risk of transmission. Despite significant progress in scaling up ART access, challenges such as ART non-adherence and poor clinical visit adherence persist, particularly in sub-Saharan Africa. For example, studies^2,3^ have shown that a substantial proportion of patients discontinue care within the first two years of treatment initiation, clearly indicating the need for interventions to improve retention.

To maximize the impact of HIV care interventions, a shift toward more targeted approaches is warranted, as current evidence indicates that one-size-fits-all interventions may not effectively allocate resources to those most in need. For instance, Fahey CA, Njau PF, Katabaro E, et al.^4^ found that small, short-term financial incentives improved viral suppression six months after ART initiation by 13 percentage points. However, 73% of the comparison group achieved viral suppression without the intervention, suggesting that more efficient targeting could enhance population-level impacts. In environments with scarce resources, ensuring that programs are efficiently tailored to populations at greatest risk is critical for optimizing outcomes. Evidence like this highlights the importance of identifying individuals most likely to experience poor outcomes and directing limited resources to where they can produce the greatest benefit.

The increasing implementation of electronic medical record (EMR) systems in sub-Saharan Africa, coupled with advancements in machine learning (ML) and artificial intelligence (AI), offers the potential to direct interventions more precisely.^5,6^ This is because ML and AI methods can potentially leverage HIV-related EMR data to predict which people face elevated risks of key outcomes by forecasting virologic outcomes, future missed visits, and attrition from care. As a result, healthcare providers can proactively engage at-risk individuals with supportive interventions, enhancing the efficiency of HIV care programs and maximizing the impact of limited resources. Nevertheless, existing studies using ML to predict PLHIV at risk of care disengagement have shown only moderate predictive power.

In this study, we develop a novel AI model by enhancing a pre-trained large language model (LLM), specifically Llama 3.1^7^–an open-source pre-trained LLM released by Meta, using routinely collected EMR data from Tanzania’s National HIV Care and Treatment Program to predict PLHIV who are at risk of disengaging from care (i.e., becoming ART non-adherent or lost-to-follow-up) or experiencing adverse clinical outcomes (i.e., having an unsuppressed viral load). Since the introduction of GPT-3 in 2020, LLMs have emerged as transformative tools with the potential to revolutionize healthcare due to their ability to process and understand large volumes of textual data and capture complex patterns within it.^8,9^ Unlike traditional ML, which often requires structured data and extensive feature engineering, LLMs can leverage unstructured data, natural language inputs, and extensive training knowledge base, enabling them to uncover nuanced relationships and develop context-specific insights. To our knowledge, this is the first study to develop an enhanced LLM based AI tool to predict PLHIV at risk of disengagement or developing adverse outcomes using large-scale EMR data collected in sub-Saharan Africa, along with novel insights into individual risk profiles that could inform targeted follow-up interventions.

## Methods

### Methods for developing the enhanced LLM; Figure 1 for method overview.

#### Data source and ethical statement

We utilizeddata from two regions in Tanzania from the national EMR repository, which includes digitized medical records manually entered after clinical visits and maintained by the Ministry of Health (MoH) in Tanzania and the National AIDS, STIs, and Hepatitis Control Programme (NASHCoP), which are referred to as CTC3 medical records. Our analysis adopted the EMRs from January 1, 2018, to June 30, 2023, resulting in 4,809,765 records for 261,192 PLHIV. These medical records include 99 variables related to demographics, clinical visit details, biomarkers and lab tests, medical history, and health facility information.

The Tanzania National Institute for Medical Research (NIMR) and the Committee for Protection of Human Subjects at the University of California, Berkeley (UC Berkeley) provided ethical review and approval for this study (UC Berkeley ethics approval number: 2021-10-14723, renewed on October 23,2024; [Tanzania NIMR ethics approval number: NIMR/HQ/R.8a/Vol.X/3992, obtained on 5 May 2022.]). The study protocol titled, “Strengthening the continuity of HIV care in Tanzania with economic support” received expedited review and approval. With approval from both ethical review boards, we received a waiver of written informed consent for this part of the study in accordance with FDA guidelines according to 45 CFR 46·116(f). Specific permission was granted to use routinely collected demographic and clinical information, as well as the minimal amount of identifiable private information to identify participants for potential future trials. These EMRs are used to enhance the pre-trained open-source LLM, LLaMA 3.1 (8-billion-parameter version of Meta’s pre-trained open-source large language model), which has been securely downloaded to our GPU cluster. The cluster is housed within UC Berkeley’s campus data center, with full encryption implemented to ensure the highest level of data protection.

#### Predictors

To ensure the practicality of our enhanced LLM model, we employed a bi-directional, participatory approach to predictor selection–this collaborative process involved close coordination with local clinicians and MoH representatives, drawing on CTC3 medical records. The selected predictors listed in **Table 1** were categorized into six groups: (1) Demographics (sex, age, marital status), (2) Clinical visit details (appointment date, visit date, visit type, client category), (3) Medical history (number of pills, duration on ART, time since HIV diagnosis, tuberculosis preventive therapy), (4) Biomarkers (antiretroviral treatment status, CD4 count, nutrition status, pregnancy status, WHO stage, viral load measurements, weight, family planning), and (5) Health facility information (facility type). To ensure relevance to current practice, we used medical records from January 1, 2018, to December 31, 2021, when constructing the predictor set.

#### Outcomes

Aiming to guide the targeted implementation of proactive interventions for PLHIV to improve retention in care, reduce morbidity, and prevent onward transmission, we define a comprehensive composite outcome using data from January 1, 2022, to June 30, 2023 (**Table 2**). This composite outcome includes three components: (1) the risk of ART non-adherence, determined by the number of days not on ART (details below); (2) high viremia, defined as a viral load exceeding 1,000 copies/mL at least once; and (3) lost-to-follow-up (LTFU), defined as missing a scheduled clinical visit by 28 days or more (following the PEPFAR^10^ definition of LTFU from facility-based care). The ART non-adherence risk profile is calculated based on the duration a patient was off ART during the study period. Using data from the CTC3 medical records, which include scheduled visit dates, actual visit dates, and the quantity of ARV pills dispensed at each clinical visit, we estimated the number of days each patient was off ART from January 1, 2022, to June 30, 2023. Patients off ART for fewer than 28 days are classified as “no risk,” aligning with the PEPFAR definition. Those off ART for more than 28 days are categorized into three risk groups based on the distribution of the number of days off ART: low risk (28 to less than 33 days, below the 25th percentile), moderate risk (33 to less than 44.5 days, between the 25th and 50th percentiles), and high risk (44.5 days or more, above the 50th percentile).

#### Fixed formatting prompt instruction

Fixed formatting and task-specific instructions are applied to facilitate downstream validation. The LLM was instructed as follows:

“As a question-answering agent, your task is to predict if people living with HIV (PLHIV) have a detectable viral load, is at risk of ART non-adherence, or will be lost to follow-up for more than 28 days in year 2022-2023. The prediction result can be formalized into an output sentence in the following format: This PLHIV will have [suppressed/detectable/unknown] viral load, is [possibly/unlikely] to be lost to follow-up for over 28 days, and is at [high/moderate/low/no/unknown]risk of ART non-adherence based on their days not on ART in the year 2022-2023. Give me your reasonings for outcome prediction.”

This instruction guides the LLM in generating outcomes in a standardized format, using predefined language to ensure accuracy and focus. This approach also allows us to extract token probabilities for specific components of predictions, which enables the application of classical multi-class classification metrics to validate model performance.

#### Natural language description of predictors and outcomes in prompt input and outcome

To adapt pre-trained LLMs for processing longitudinal EMRs, we performed feature engineering to convert predictors and outcomes into natural language–a process known as textual serialization.^11–14^ This conversion makes the data compatible with LLMs, which are inherently designed to process textual information. Table 1 details the serialization process for the predictors extracted from the CTC3 medical records. Our serialization approach varied depending on the type of variable. Some variables, such as demographics (e.g., sex, age), were converted into natural language formats. In contrast, others, like clinical visits and biomarker data, were serialized as structured text sequences (e.g., dates, and weight measurements). Baseline demographics and health facility information were serialized into interpretable text-based formats, enabling the LLM to process tabular data effectively. Longitudinal variables, such as clinical visit data and biomarkers, were transformed into narrative sequences that preserve the temporal dynamics essential for modeling patient health trajectories. Missing data was represented as “NA,” allowing the LLM to interpret the absence of information without requiring imputation. See **Table 2** for details following the prompt instruction.

#### Utilizing summarized longitudinal predictors

To capture temporal trends in longitudinal EMRs, we instructed LLaMA 3.1 to generate narrative summaries of individual patient predictors with the following prompt: “*Please review the patient’s medical records from 2018 to 2021. For example, the summary could include how patient profiles change over the years, periods of missed or delayed appointments, changes or gaps in follow-up care related to ART adherence, patterns in the number of pills dispensed during this time, and fluctuations in CD4 count, viral load, or weight*.”

#### Enhance pre-trained LLM with fine-tuning

To improve the predictive accuracy of a pre-trained LLM in predicting comprehensive outcomes for PLHIV, we fine-tuned LLaMA 3.1 using fixed formatting instruction and natural language descriptions of predictors and outcomes as input training prompts. Fine-tuning involves adapting a pre-trained LLM with a specialized dataset, enhancing its ability to perform specific tasks while minimizing computational demands. In this study, we used Low-Rank Adaptation (LoRA),^15^ an efficient method for fine-tuning that optimizes only a subset of model parameters. We developed two fine-tuned LLMs using the same LoRA-based approach, with differences in the structure and focus of their input data. Both models were trained with a fixed-format prompt instruction, guiding the LLM to predict the composite outcome comprising three components (the risk of ART non-adherence, non-suppressed viral load, and LTFU). The first model utilized only the generated longitudinal summaries of patient data as inputs. In contrast, the second model incorporated both the longitudinal summaries and detailed natural language descriptions of the predictors. While the underlying prediction task remained consistent across both models, the second model was designed to leverage the richer contextual information embedded in the expanded input structure, enabling a more nuanced assessment of patient outcomes.

### Methods for evaluating, validating, and interpreting the enhanced LLMs

#### External and internal validation datasets

The enhanced LLMs underwent both internal and external validation. For internal validation, 80% of the CTC3 medical records from Kagera region from January 1, 2022, to June 30, 2023 were randomly selected for model development, with the remaining 20%, which were completely withheld during training, used for validation. The model trained on 80% of CTC3 data from Kagera region was then externally validated on CTC3 data from Geita region. Summary distributions of predictors in both the training and validation datasets are provided in Table 3.

#### Evaluation metrics

We compared the performance of our enhanced LLM with a state-of-art ML method^16,17^ and zero-shot LLaMA 3.1. For the supervised ML method, we employ the gradient boosting method, an ensemble learning method known to have robust performance in prediction tasks. Specifically, we utilize the *CatBoost*^18^Python package that implements the gradient boosting method while optimizing computational efficiency. For the zero-shot method, we instructed LLaMA 3.1 to make predictions directly based on natural language descriptions of patient predictors and outcomes.

Our prompt instructions specified a fixed format for prompt outputs, facilitating easy extraction of token probabilities and enabling us to apply classical methods to evaluate model performance. We assessed the performance of various models using three metrics: the classical Area Under the Receiver Operating Characteristic Curve (AUC), effort-benefit trade-off curves, and subgroup analyses. Receiver operating characteristic (ROC) curves were plotted to visualize the trade-off between true positive rates (TPR, sensitivity) and false positive rates (1 – specificity) at various thresholds. For the multi-class outcome variable, we reported multi-class AUC^19^ using a one-vs-rest approach. Effort-benefit trade-off curves plot the cumulative TPRs, representing the proportion of correctly identified at-risk patients, against the proportion of PLHIV predicted to be at highest risk of LTFU–defined as those with calculated risk scores exceeding a given threshold--who can be prioritized for proactive support interventions under resource constraints. Lastly, subgroup analyses examined how model performance varied across different patient groups as defined by age and sex.

#### Attention scores for model interpretation

Attention scores are weights in a language model assigned to each token in the input sequence, indicating the relative importance of each token when predicting the output.^20,21^ Higher attention scores often imply that a group of tokens is more influential for the models prediction. See technical details for attention score extraction in supplements.

#### Human expert evaluation

Two local HIV physician researchers in Tanzania evaluated the prediction results of the enhanced LLM for 20 patient medical records. They independently reviewed the LLM-summarized longitudinal patient medical records (a synthetic summary provided in the Supplementary Materials to ensure patient privacy) to predict the patient LTFU status. Additionally, they were provided with the enhanced LLM’s justifications and assessed whether the reasoning was reasonable and aligned with the information in the reviewed medical records.

## Results

Table 3 summarizes the multi-class AUC values for predicting the risk of ART non-adherence category, viral suppression, and LTFU using different models on internal (Kagera region 20% subset) and external (Geita region) validation sets. The enhanced LLM fine-tuned with both summarized and natural language descriptions of EMRs consistently achieved the highest AUCs across all outcomes. For the risk of ART non-adherence category, it attained AUCs of 0.74 (internal) and 0.67 (external). The enhanced LLM using only summarized data closely followed with AUCs of 0.73 (internal) and 0.66 (external), while the supervised machine learning model yielded AUCs of 0.72 (internal) and 0.66 (external).The zero-shot LLMs performed significantly worse, with AUCs of 0.53–0.55 (internal) and 0.54–0.55 (external). In predicting viral suppression, the enhanced LLM with both data types achieved AUCs of 0.68 (internal) and 0.64 (external), outperforming the summary-only LLM (0.65 internal; 0.62 external) and the supervised model (0.68 internal; 0.64 external). The zero-shot LLMs had AUCs around 0·51 for both validation sets. For LTFU, the enhanced LLM with combined inputs achieved the highest AUCs of 0.77 (internal) and 0.71 (external). The summary-only LLM followed with AUCs of 0.76 (internal) and 0.68 (external), and the supervised model obtained 0.71 (internal) and 0.65 (external). Zero-shot LLMs lagged with AUCs of 0.55–0.57 across both sets. Overall, the enhanced LLMs, especially when fine-tuned with both summarized and natural language descriptions of EMRs, outperformed the supervised machine learning model and zero-shot LLMs across all outcomes.

**Figure 2:**
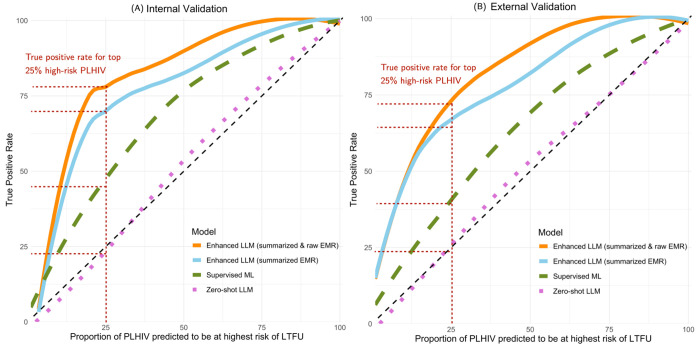
Effort-benefit trade-off curves describes the cumulative TPRs (the proportion of correctly identified at-risk patients) against the proportion of PLHIV predicted to be at highest risk of LTFU who can be prioritized for proactive support interventions within resource constraints.

The effort-benefit trade-off curves in Figure 2 illustrate the cumulative TPR against the proportion of PLHIV predicted to be at highest risk of LTFU who can be prioritized for proactive support interventions. The enhanced LLM using both summarized and natural language descriptions of EMRs consistently achieved the highest cumulative TPR across all proportions. For instance, when defining 25% of patients identified as will be LTFU, this model achieved TPRs of 78% in internal validation and 73% in external validation. The LLM using only summarized data also performed well, achieving high TPRs (72% and 64% for internal and external validation). In comparison, the supervised ML exhibited lower cumulative TPRs across both internal (46%) and external (39%) validation, and the zero-shot LLM had the lowest TPR values (22% and 21% for internal and external validation).

**Figure 3:**
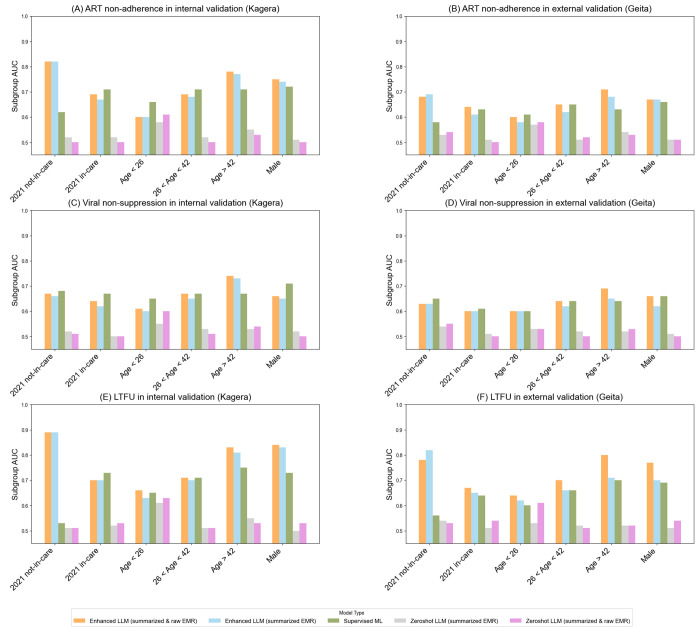
Subgroup analysis of AUC comparisons across different in-care, age and sex groups.

The subgroup analysis in Figure 3 shows that the enhanced LLM using both summarized and natural language descriptions of EMRs achieved the highest AUC across all subgroups, particularly excelling in patients not in care in 2021 and those over 42 years of age, with AUCs up to 0.89 for internal and 0·80 for external validation in predicting LTFU. The LLM using summarized data alone also performed well, though slightly lower, achieving similar scores for patients not in care but with reduced performance for younger age groups. The supervised ML model performed moderately, achieving AUCs up to 0.75 for various age groups, but it struggled with LTFU prediction for patients not in care (AUCs ranging 0.53–0.56). Zero-shot LLMs performed poorly across all subgroups, with AUCs generally below 0.55.

**Figure 4:**
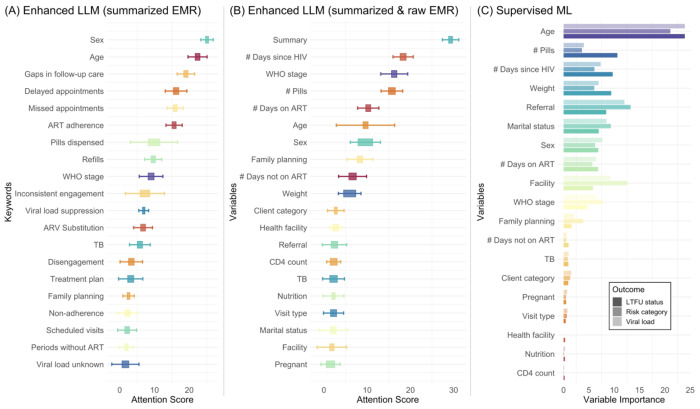
Attention scores of enhanced LLMs and feature importance for supervised ML.

Figure 4 presents the attention scores of enhanced LLMs and feature importance for the supervised ML (gradient boosting) model. For the enhanced LLM using summarized EMRs (Panel A), the highest attention scores were given to the keywords “gaps in follow-up care,” “delayed appointments,” and “ART adherence,” suggesting that these factors are most critical for predicting defined HIV risk outcomes. In the enhanced LLM using both summarized and raw EMR data (Panel B), similar variables were highlighted, with the “summary” feature receiving the highest attention, indicating that the summarized information played a crucial role in model prediction. Additionally, key words like “# days on ART” and “WHO stage” also received high attention scores, emphasizing their importance for accurate prediction. The supervised ML model (Panel C) showed the highest variable importance for “age” and “# pills,” followed by “# days since HIV diagnosis” and “weight.”

Lastly, the agreement between human and LLM decisions—defined as both arriving at the same prediction regarding patient LTFU status—was observed in 65% of cases (13 out of 20). When aligned, Tanzanian HIV care providers found the AI-provided justifications to be reasonable, with 92.3% being rated as sensible. In cases where physician and AI decisions were misaligned (7 out of 20), the AI provided correct answers 57% of the time (4 out of 7), while humans provided correct answers in 43% of those cases (3 out of 7). However, in instances of misalignment, experts judged the LLM’s justifications as not helpful.

## Discussion

We developed and validated two enhanced LLMs based on LLaMA 3.1, fine-tuned with EMRs from Tanzania’s National HIV Care and Treatment Program, to predict PLHIV at risk of ART non-adherence, LTFU, and developing non-suppressed viral load. Our findings demonstrate that the enhanced LLMs, particularly when utilizing both summarized and natural language descriptions of EMR data, significantly outperform traditional supervised ML models and zero-shot LLM in all outcomes. Our enhanced LLM not only achieved higher AUC values but also maintained robust performance across various subgroups, suggesting broad applicability in diverse patient populations.

Compared to previous studies using ML to predict HIV care disengagement, our enhanced LLMs demonstrated both superior predictive performance and the ability to address multiple clinically meaningful HIV outcomes within a single framework. For example, prior ML-based approaches have typically focused on a single outcome. Esra RT, Carstens J, Estill J, et al.^22^ developed an ML model in South Africa to predict subsequent missed ART visits, achieving a sensitivity of 61.9% and a PPV of 19·7%, while Maskew M, Sharpey-Schafer K, De Voux L, et al.^23^ applied ML algorithms in two South African districts to predict the risk of LFTU, reporting an AUC of 0.68. Similarly, Xie Z, Hu H, Kadota JL, et al.^17^ leveraged EMR data from two regions in Tanzania to forecast LTFU, achieving an AUC of 0.65. When we compare the performance on the same outcome of LTFU, our LLMs achieved more powerful predictive performance, with AUCs of 0.77 for internal validation and 0.71 for external validation, surpassing the results reported by both Maskew M, Sharpey-Schafer K, De Voux L, et al.^23^ (AUC=0.68) and Xie Z, Hu H, Kadota JL, et al.^17^ (AUC=0.65). This combination of improved predictive power and a broader scope of clinically relevant outcomes demonstrate the unique potential of LLM-based methods to advance HIV care.

Our findings have potential implications for HIV care programs in sub-Saharan Africa. By accurately identifying patients at higher risk of disengagement or adverse outcomes, healthcare providers can allocate resources more efficiently and implement targeted interventions to groups who are most in need. For example, when focusing on the top 25% of patients identified by our model as will be LTFU, we found that 85% of these people in internal validation and 80% in external validation were genuinely LTFU (Figure 2). This high level of accuracy enables healthcare teams to concentrate their efforts on a smaller subset of PLHIV who are most likely to benefit from additional support, thereby maximizing the impact of limited resources. In addition, our enhanced LLM works in a manner akin to a clinician by reading patient summaries, providing risk predictions, and offering reasoning for its assessments. In the future, such AI models could be integrated into clinical workflows to collaborate side by side with human experts, augmenting their ability to monitor patient progress and intervene promptly when necessary. This human-AI collaboration not only facilitates more informed clinical evaluations and decision-making but also aligns with the UNAIDS “95-95-95” targets, potentially contributing to reduced HIV transmission and improved health outcomes for PLHIV.

An innovative technical aspect of our enhanced LLM is the use of textual summaries of longitudinal EMRs, which not only improved the model’s predictive performance but also provided a valuable tool for HIV clinicians to monitor patient health and to provide timely interventions. Traditionally, clinicians must sift through large volumes of patient medical records, a task that is both time-consuming and prone to oversight. By providing interpretable summaries, our model helps physicians quickly identify critical changes and emerging health issues, effectively augmenting their capacity to manage complex individual medical histories. Additionally, whereas conventional ML approaches may appear as “black boxes” that are difficult to interpret, our LLMs provide the underlying reasoning and temporal patterns captured by the model. Compared to LLMs, the ML model relied more on demographic and treatment duration features (Figure 4C). The enhanced LLMs, especially with the summarized feature included, appeared to focus more on care continuity and adherence-related aspects, highlighting their nuanced understanding of patient engagement in HIV care (Figure 4A). The LLM’s dual focus on interpretability and clinical relevance enables timely interventions and supports more informed decision-making. Independent evaluations by clinical experts confirm that these summaries are both clinically accurate and highly actionable, illustrating the potential of this technique to advance HIV care and other chronic conditions requiring sustained engagement.

Our results suggest that LTFU, as defined by a 28 day or greater disruption in care, may be the most actionable outcome for identifying higher risk individuals who can be reached with personalized HIV interventions, as it achieved the highest predictive performance across all models under comparison. Recall that the enhanced LLM reached AUC up to 0.77 in internal validation and 0.71 in external validation for LTFU prediction, surpassing its performance on other outcomes like risk category and viral non-suppression. The ability to accurately predict patients who will be LTFU is valuable, as it enables healthcare providers to implement timely interventions aimed at retaining patients in care, thereby improving adherence to antiretroviral therapy before the patient experiences viral rebound, thereby reducing the risk of onward transmission.

Despite these promising results, there are limitations to consider. While the enhanced LLMs outperformed traditional ML models, there remains room for model further improvement. Future research could explore integrating additional data sources, such as social determinants of health or patient-reported outcomes, to enhance model performance. Additionally, our study utilized data from two regions in Tanzania, and although we conducted external validation, the generalizability to other settings may be limited. Evaluating the models in different geographic and healthcare contexts is necessary to confirm their broader applicability. Currently, the model is hosted locally at UC Berkeley in a secure data environment, ensuring robust data privacy protections. Looking ahead, our private LLM environment at Berkeley provides a secure platform for expanding these capabilities to other parts of sub-Saharan Africa. This effort will require robust data-sharing agreements, strict adherence to privacy regulations, and strong encryption measures to safeguard patient information.

In conclusion, this study demonstrates the potential of enhanced LLMs in predicting the risk profiles of PLHIV using large-scale EMR data and in supporting clinicians through interpretable summaries. The improved predictive performance over traditional ML models suggests that advanced AI approaches can play a pivotal role in optimizing future HIV care. Future research could focus on refining these models, integrating them into clinical workflows, and assessing their impact on patient outcomes.

## Figures and Tables

**Figure 1:**
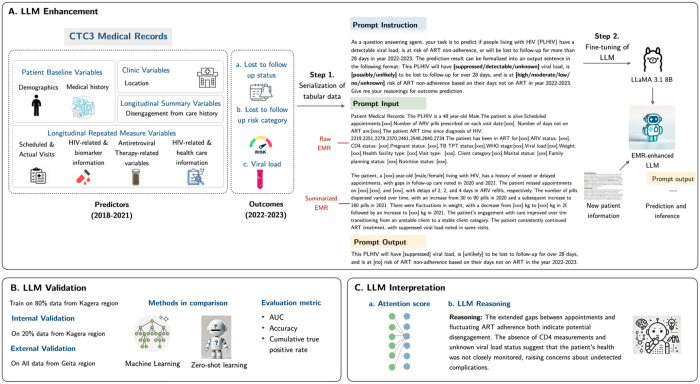
Method for open-source language model (LLaMA 3.1) enhancement pipeline. Specific words are highlighted in red to illustrate how standardization of language and format reduces model hallucination and improves prediction consistency.
